# A TyG–UHR-based machine learning model for screening lean MAFLD: development and external validation

**DOI:** 10.1186/s12938-026-01585-8

**Published:** 2026-05-11

**Authors:** Yansong Ji, Hui Zhang, Jingjing Xia, Guilian Chen, Hong Li

**Affiliations:** 1 Health Management Center, the Affiliated Huai’an No.1 People’s Hospital of Nanjing Medical University, Huai’an, 223300 China; 2 Department of Neurology, Huai’an Hospital Affiliated to Yangzhou University, Huai’an, 223300 China

**Keywords:** Lean MAFLD, Health checkup, TyG, UHR, Machine learning, Calibration, Decision curve analysis, SHAP, External validation

## Abstract

**Background:**

Lean metabolic dysfunction-associated fatty liver disease (MAFLD) is increasingly recognized but often goes unnoticed during health checkups and primary care due to low perceived risk and limitations of imaging. Cost-effective, automated screening methods using routine data could enhance triage and referrals for confirmatory imaging.

**Methods:**

We used routinely collected data from a hospital health checkup cohort (*N* = 3346; Huai’an First People’s Hospital, China) to develop a machine learning model for lean MAFLD incorporating prespecified predictors––triglyceride-glucose (TyG) and uric acid–to–high-density lipoprotein cholesterol ratio (UHR). The cohort was randomly split 70:30 into training and test sets. In the training set, additional clinical predictors were selected via elastic-net regression, followed by the tuning of eight algorithms with strict leakage control, utilizing average precision as the primary optimization metric. Extreme gradient boosting (XGBoost) was chosen based on test set discrimination, calibration, and clinical utility. External validation utilized data from the National Health and Nutrition Examination Survey 2017–2020 (*N* = 2216), split 50:50 into calibration and hold-out validation subsets. Post hoc recalibration was performed on the calibration subset. Performance in the hold-out validation subset was summarized using the receiver operating characteristic curve, calibration plots, decision curve analysis, and Shapley Additive Explanations (SHAP) for model interpretability.

**Results:**

Restricted cubic spline analysis revealed a nonlinear association for TyG, whereas UHR showed an almost linear association. Elastic-net regularization identified additional clinical predictors, including systolic blood pressure (SBP), diastolic blood pressure (DBP), age, the alanine aminotransferase/aspartate aminotransferase (ALT/AST) ratio, and serum creatinine (Scr). In the test set, XGBoost achieved an area under the curve (AUC) of 0.995, a Brier score of 0.008, a log loss of 0.035, and an expected calibration error of approximately 0.007, offering the highest net benefit across threshold probabilities of 0.05 to 0.60. In the hold-out validation subset, discrimination remained robust both before and after recalibration (AUC 0.826 vs. 0.810), while probability accuracy improved significantly (Brier score decreased from 0.286 to 0.109; log loss from 1.253 to 0.388). Recalibrated probabilities demonstrated acceptable calibration (intercept 0.103; slope 0.684), with consistent positive net benefit observed across thresholds of approximately 0.02 to 0.30. Regarding interpretability, SHAP values ranked TyG (2.538) as the most influential factor, followed by SBP (1.727) and UHR (0.999), with modest but consistent contributions from age (0.810), Scr (0.713), and the ALT/AST ratio (0.657), whereas DBP (0.266) had the smallest effect.

**Conclusions:**

An explainable machine learning model centered on TyG and UHR can cheaply screen for lean MAFLD using routine health checkup data, support threshold-based triage in primary care, and be adapted to external populations with post hoc recalibration. Adoption should include prediction paired with lightweight local recalibration to maintain accuracy and make implementation easier across different sites.

**Supplementary Information:**

The online version contains supplementary material available at 10.1186/s12938-026-01585-8.

## Introduction

Metabolic dysfunction-associated fatty liver disease (MAFLD) is now the most common chronic liver disease worldwide, and its burden continues to grow in both prevalence and incidence, including in China [1, 2]. The new terminology focuses on metabolic dysfunction, framing hepatic steatosis as a systemic metabolic disorder rather than just an accumulation of fat in the liver [3–5]. This approach better aligns clinical evaluation with the underlying pathophysiology and with population-level trends in metabolic risk [6, 7].

Although MAFLD is strongly associated with excess adiposity, a significant portion of patients are lean [8]. Meta-analyses suggest that, in the general population, lean MAFLD affects about 2% [8], while among lean adults defined by region-specific body mass index (BMI) thresholds, the prevalence is roughly 5% [9]. For the earlier lean nonalcoholic fatty liver disease (NAFLD) definition, pooled prevalence in lean individuals is around 9–10%, and approximately 20% of all NAFLD cases occur in people without obesity [10]. These estimates vary depending on the diagnostic method—such as ultrasound, magnetic resonance imaging (MRI), vibration-controlled transient elastography (VCTE) with controlled attenuation parameter (CAP), or biochemical indices—and the BMI cutoffs used in Asian and Western populations [8, 11], but consistently show that lean individuals are not immune to metabolically driven fatty liver [12].

In routine health checkups and primary care, lean or low-BMI adults are rarely targeted for MAFLD testing because their risk is often considered minimal. Meanwhile, imaging-based methods face practical limitations: ultrasound is labor-intensive and operator-dependent, computed tomography (CT) exposes patients to ionizing radiation, and MRI—despite being accurate—has limited throughput and high costs [13]. As a result, lean MAFLD is probably underdetected, which delays counseling and cardiometabolic risk management.

Therefore, there is a need for affordable, automatable, and scalable screening tools that can be integrated into health screening workflows. Two easily accessible metrics show promise for this purpose: the triglyceride-glucose (TyG) index, a validated surrogate for insulin resistance (IR) [14], and the uric acid–to–high-density lipoprotein cholesterol ratio (UHR), which reflects combined signals related to purine metabolism, inflammation, and lipid regulation [15, 16]. Both are low-cost, routinely measured, and derivable from standard lab panels, making them attractive inputs for digital screening. Modern machine learning techniques can combine TyG, UHR, and a small set of routine clinical variables to generate calibrated, individual risk estimates suitable for triage. When developed with strict control of data leakage, evaluated for discrimination, calibration, and decision curve analysis (DCA), and accompanied by transparent explainability, these models can offer actionable, threshold-based guidance on referrals for confirmatory imaging.

We aimed to develop and externally validate a machine learning-based screening model for lean MAFLD using TyG, UHR, and routine clinical indicators available in health checkups and primary care. We trained the model on a regional health checkup cohort and tested its generalizability in an independent U.S. population sample from the National Health and Nutrition Examination Survey (NHANES) 2017–2020, evaluating discrimination, calibration, and clinical utility across plausible screening thresholds. Although recent proposals introduced the term metabolic dysfunction-associated steatotic liver disease [4], we followed the 2020 MAFLD consensus definition for case adjudication because it aligns with our clinical workflows and allows seamless deployment in health check settings [17].

## Results

### Baseline characteristics

In the hospital cohort, participants with lean MAFLD were older (52.74 ± 12.92 vs. 37.00 ± 11.50 years; *P* < 0.001) and more likely to be male (55.15% vs. 20.39%; *P* < 0.001) than controls. They also had higher blood pressure—systolic blood pressure (SBP) 133 ± 18 vs. 111 ± 10 mmHg and diastolic blood pressure (DBP) 80 ± 11 vs. 67 ± 7 mmHg (both *P* < 0.001)—worse glycemic indices—fasting blood glucose (FBG) 5.34 (4.95–6.12) vs. 4.72 (4.47–4.98) mmol/L and glycated hemoglobin A1c (HbA1c) 5.85 (5.50–6.40) vs. 5.30 (5.18–5.50)% (both* P* < 0.001)—and a more atherogenic lipid profile—triglycerides (TG) 1.87 (1.43–2.61) vs. 0.80 (0.63–1.02) mmol/L, high-density lipoprotein cholesterol (HDL-C) 1.21 ± 0.29 vs. 1.64 ± 0.29 mmol/L, and low-density lipoprotein cholesterol (LDL-C) 3.06 ± 0.85 vs. 2.68 ± 0.70 mmol/L (all *P* < 0.001). Liver enzymes were also higher in lean MAFLD: alanine aminotransferase (ALT), 20 (15–27) vs. 12 (9–16) U/L (*P* < 0.001), and aspartate aminotransferase (AST), 20 (17–24) vs. 17 (15–20) U/L (*P* < 0.001). Renal markers were elevated as well—UA 323.20 ± 80.70 vs. 261.88 ± 63.63 μmol/L and blood urea nitrogen (BUN) 5.32 ± 1.36 vs. 4.82 ± 1.23 mmol/L (both *P* < 0.001)—and serum creatinine (Scr) was slightly higher (65.92 ± 15.10 vs. 61.10 ± 11.49 μmol/L; *P* < 0.001). As predefined indices, TyG (9.11 ± 0.60 vs. 8.01 ± 0.36) and UHR (12.47 ± 4.85 vs. 7.25 ± 2.54) were significantly elevated in lean MAFLD (both *P* < 0.001), while lipoprotein(a) [Lp(a)] showed no substantial difference (*P* = 0.057). In the external dataset, results were directionally similar: lean MAFLD participants were older (59.30 ± 15.58 vs. 43.12 ± 19.70 years; *P* < 0.001), exhibited higher SBP and DBP (both* P* < 0.001), elevated FBG and HbA1c (both* P* < 0.001), increased TG, and decreased HDL-C (both *P* < 0.001), in addition to higher ALT and AST levels (both *P* < 0.001). UA and BUN levels were also increased (both *P* < 0.001), while Scr did not significantly differ (*P* = 0.073). Additional measures in the external dataset—homeostatic model assessment of insulin resistance (HOMA-IR) and C-reactive protein (CRP)—were also elevated in lean MAFLD (both *P* < 0.001). Refer to Table 1 for detailed data.

**Table 1:** Baseline characteristics of the hospital health checkup cohort and the NHANES 2017–2020 external dataset

Variable	Hospital cohort		*P* value	External dataset (NHANES 2017–2020)		*P* value
	Lean MAFLD (*n* = 330)	Controls (*n* = 3016)		Lean MAFLD (*n* = 345)	Controls (*n* = 1871)	
Age	52.74 ± 12.92	37.00 ± 11.50	< 0.001	59.30 ± 15.58	43.12 ± 19.70	< 0.001
Sex			< 0.001			0.080
Male	182 (55.15%)	615 (20.39%)		182 (52.75%)	888 (47.46%)	
Female	148 (44.85%)	2401 (79.61%)		163 (47.25%)	983 (52.54%)	
SBP	133 ± 18	111 ± 10	< 0.001	132 ± 20	120 ± 19	< 0.001
DBP	80 ± 11	67 ± 7	< 0.001	75 ± 12	70 ± 11	< 0.001
FBG	5.34 (4.95–6.12)	4.72 (4.47–4.98)	< 0.001	5.38 (5.00–6.11)	4.88 (4.61–5.22)	< 0.001
HbA1c	5.85 (5.50–6.40)	5.30 (5.18–5.50)	< 0.001	5.80 (5.40–6.30)	5.30 (5.10–5.60)	< 0.001
TC	4.97 ± 0.99	4.51 ± 0.79	< 0.001	5.10 ± 1.22	4.62 ± 1.01	< 0.001
TG	1.87 (1.43–2.61)	0.80 (0.63–1.02)	< 0.001	1.58 (1.05–2.29)	0.91 (0.69–1.25)	< 0.001
HDL-C	1.21 ± 0.29	1.64 ± 0.29	< 0.001	1.41 ± 0.44	1.60 ± 0.44	< 0.001
LDL-C	3.06 ± 0.85	2.68 ± 0.70	< 0.001	NA	NA	NA
Lp(a)	153.80 (101.70–248.22)	163.10 (104.47–311.27)	0.057	NA	NA	NA
ALT	20 (15–27)	12 (9–16)	< 0.001	18 (14–24)	14 (11–19)	< 0.001
AST	20 (17–24)	17 (15–20)	< 0.001	21 (17–24)	19 (16–23)	< 0.001
TBil	10.60 (7.90–13.80)	10.40 (7.90–13.80)	0.508	6.80 (5.10–8.60)	6.80 (5.10–10.30)	0.111
DBil	4.12 ± 1.46	4.33 ± 1.59	0.022	NA	NA	NA
IBil	6.70 (4.90–9.07)	6.35 (4.50–8.70)	0.050	NA	NA	NA
GLB	25.83 ± 3.31	25.52 ± 3.20	0.097	31.05 ± 3.93	30.26 ± 4.31	< 0.001
ALB	47.31 ± 2.56	47.11 ± 2.46	0.170	41.61 ± 3.23	41.71 ± 3.36	0.608
ALT/AST ratio	1.06 ± 0.33	0.78 ± 0.27	< 0.001	0.95 ± 0.30	0.81 ± 0.27	< 0.001
ALP	84 ± 23	62 ± 18	< 0.001	81 ± 27	72 ± 28	< 0.001
PAB	314.21 ± 71.32	257.73 ± 53.18	< 0.001	NA	NA	NA
HBDH	130 (118–145)	122 (111–135)	< 0.001	NA	NA	NA
CK	78 (61–105)	70 (55–94)	< 0.001	NA	NA	NA
LDH	169 (156–188)	159 (144–176)	< 0.001	NA	NA	NA
GGT	25 (18–40)	12 (10–16)	< 0.001	NA	NA	NA
UA	323.20 ± 80.70	261.88 ± 63.63	< 0.001	326.93 ± 82.14	282.72 ± 76.01	< 0.001
BUN	5.32 ± 1.36	4.82 ± 1.23	< 0.001	5.68 ± 2.07	4.97 ± 1.93	< 0.001
Scr	65.92 ± 15.10	61.10 ± 11.49	< 0.001	79.50 ± 51.20	77.10 ± 41.20	0.073
Cys-C	0.82 (0.74–0.92)	0.73 (0.65–0.81)	< 0.001	NA	NA	NA
WBC	6.16 ± 1.42	5.57 ± 1.40	< 0.001	NA	NA	NA
RBC	4.82 ± 0.48	4.49 ± 0.41	< 0.001	NA	NA	NA
FT3	5.10 (4.69–5.53)	4.90 (4.49–5.29)	< 0.001	NA	NA	NA
FT4	16.60 (15.00–18.20)	16.70 (15.30–18.20)	0.209	NA	NA	NA
TSH	2.01 (1.36–3.25)	2.17 (1.63–3.08)	0.012	NA	NA	NA
TyG	9.11 ± 0.60	8.01 ± 0.36	< 0.001	8.91 ± 0.64	8.23 ± 0.48	< 0.001
UHR	12.47 ± 4.85	7.25 ± 2.54	< 0.001	10.98 ± 4.40	8.32 ± 3.62	< 0.001
HOMA-IR	NA	NA	NA	2.49 (1.55–3.70)	1.23 (0.80–1.79)	< 0.001
CRP	NA	NA	NA	1.42 (0.71–3.04)	0.77 (0.43–1.69)	< 0.001

Data are presented as mean ± SD or median (IQR) and *n* (%), as appropriate. *P* values compare lean MAFLD vs. controls using Student’s *t* test for normally distributed continuous variables, Wilcoxon rank-sum test for nonnormally distributed continuous variables, and χ^2^ test or Fisher’s exact test for categorical variables. Units are harmonized across cohorts as follows: blood pressure, mmHg; glucose and lipids, mmol/L; liver enzymes, U/L; albumin/globulin, g/L; prealbumin, mg/L; lipoprotein(a), mg/L; cystatin C, mg/L; C-reactive protein, mg/L; bilirubin, μmol/L; urea nitrogen, mmol/L; creatinine, μmol/L; uric acid, μmol/L. UHR was computed as UA (mg/dL)/HDL-C (mg/dL) × 100 after unit harmonization and is reported on a unitless percentage scale. Variables not available in a cohort are denoted as NA

### Focal factor analysis

Predictive value of TyG and UHR across models. In sequentially nested logistic regression models, both TyG and UHR were consistently and strongly associated with lean MAFLD (Table 2). In single-marker models, higher TyG was linked to greater odds of lean MAFLD (Model 1: OR = 1443.39; 95% CI = 695.64–3224.78; *P* < 0.001), and UHR was also significant (Model 2: OR = 1.48; 95% CI = 1.43–1.54; *P* < 0.001). After including both markers and adjusting for sex and age (Model 3), both remained independently associated (TyG, OR = 413.30; 95% CI = 191.24–968.93; UHR, OR = 1.28; 95% CI = 1.19–1.39; both *P* < 0.001). In the fully adjusted model that additionally considered SBP, ALT/AST ratio, Scr, TSH, and WBC (Model 4), effect sizes and significance were mostly maintained (TyG, OR = 579.67; 95% CI = 215.66–1760.46; UHR, OR = 1.38; 95% CI = 1.25–1.53; both *P* < 0.001), showing that the two markers offer independent, strong predictive information beyond standard covariates.

**Table 2:** Independent associations of TyG and UHR with lean MAFLD in nested logistic models

Variables	Model 1		Model 2		Model 3		Model 4	
	OR (95% CI)	*P* value	OR (95% CI)	*P* value	OR (95% CI)	*P* value	OR (95% CI)	*P* value
TyG	1443.39 (695.64–3224.78)	< 0.001	NA	NA	413.30 (191.24–968.93)	< 0.001	579.67 (215.66–1760.46)	< 0.001
UHR	NA	NA	1.48 (1.43–1.54)	< 0.001	1.28 (1.19–1.39)	< 0.001	1.38 (1.25–1.53)	< 0.001

Model 1: TyG only; Model 2: UHR only; Model 3: TyG and UHR adjusted for sex and age; Model 4: additionally adjusted for SBP, ALT/AST ratio, Scr, TSH, and WBC. NA indicates predictor not included in the model

**Fig. 1:**
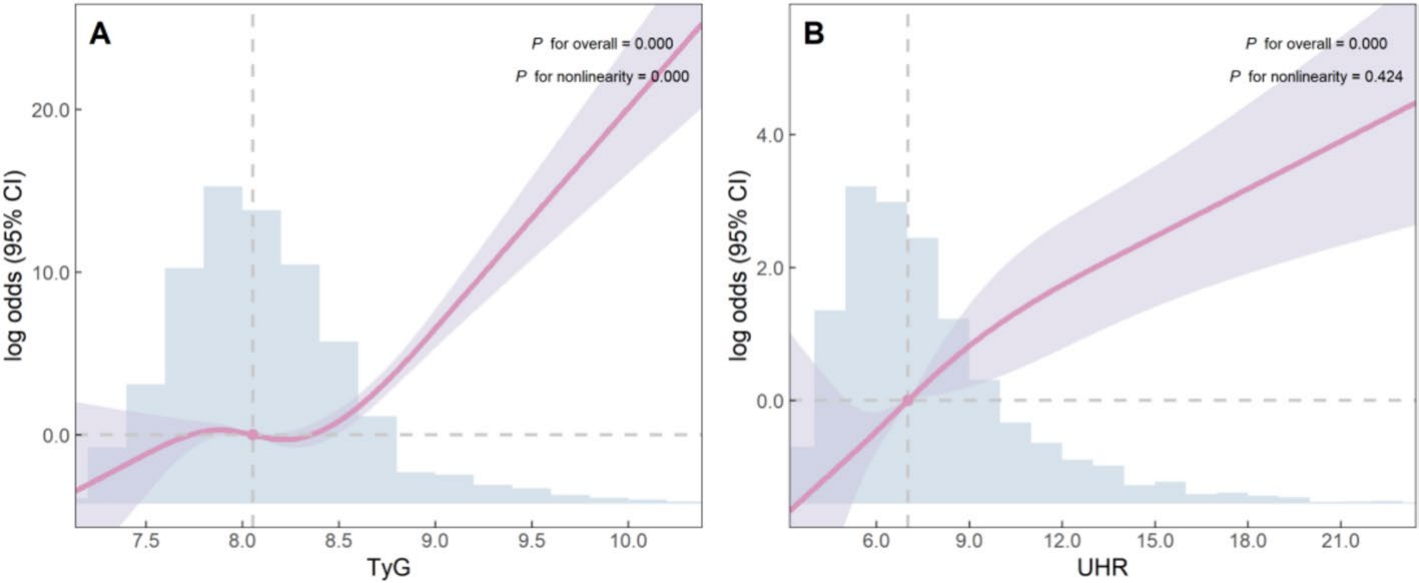
RCS associations of TyG and UHR with lean MAFLD in the fully adjusted model. **A** TyG and **B** UHR fitted with 4-knot RCS in a logistic model adjusted for sex, age, SBP, ALT/AST ratio, Scr, TSH, and WBC. Solid lines show estimated log-odds; shaded bands, 95% CIs. Histograms depict marker distributions; vertical dashed lines indicate median (reference) values

Restricted cubic splines (RCS) dose–response patterns (Model 4). In the fully adjusted model, RCS showed a significant nonlinear relationship between TyG and lean MAFLD (*P* for overall < 0.001; *P* for nonlinearity < 0.001): risk remained relatively flat at lower values and increased sharply beyond approximately 8.5. In contrast, the UHR association was roughly linear (*P* for overall < 0.001; *P* for nonlinearity = 0.424), with odds steadily rising across the range (Fig. 1).

### Selection of additional clinical predictors

**Fig. 2:**
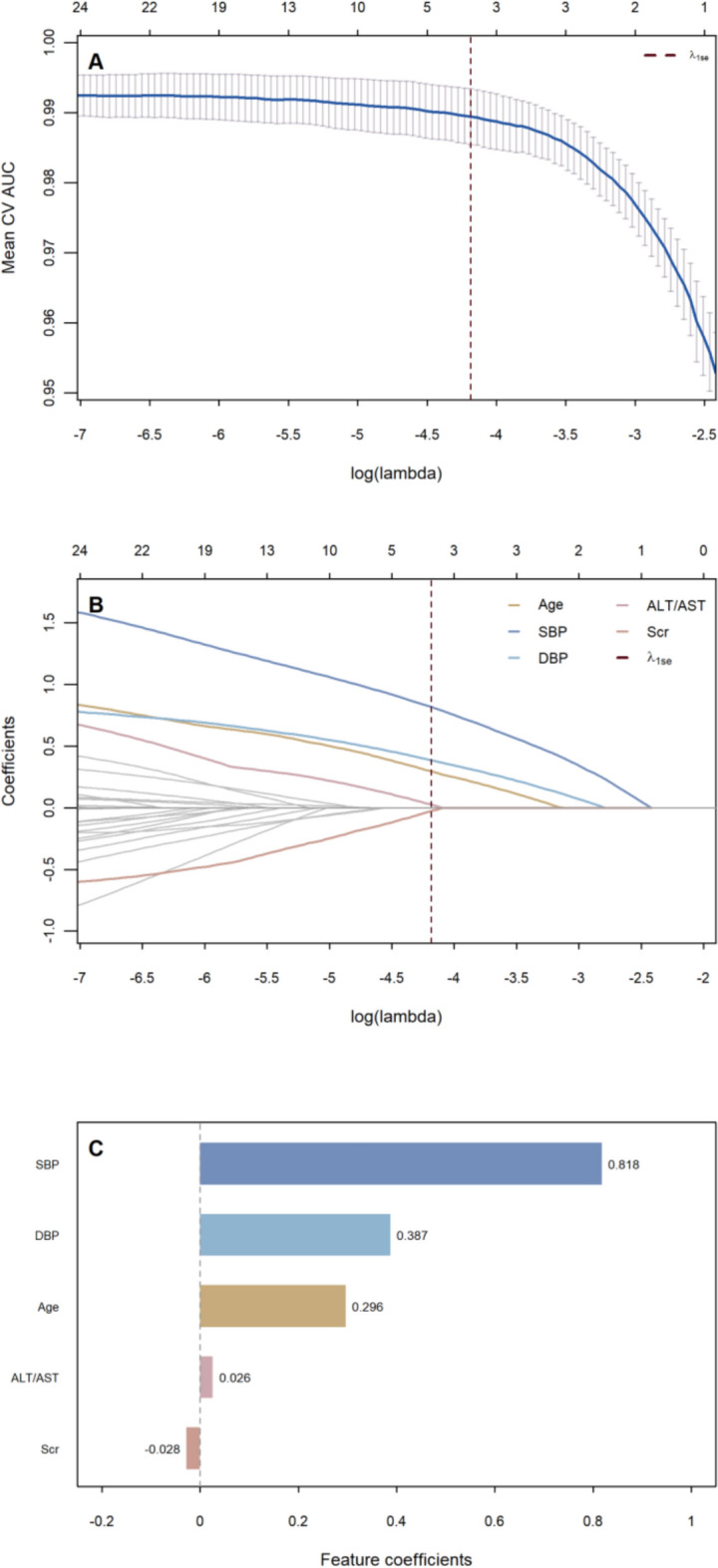
Elastic-net-based selection of additional clinical predictors beyond TyG and UHR. **A** Cross-validated AUC as a function of log(λ); the dashed vertical line marks λ₁ₛₑ (= 0.01528), and error bars denote mean ± SD across the five cross-validation folds. **B** Coefficient paths for candidate predictors across log(λ). **C** Standardized coefficients of the nonzero supplemental predictors at λ₁ₛₑ

We employed elastic-net regression to enhance TyG and UHR by identifying additional clinical predictors. As shown in Fig. 2A, at the optimal regularization parameter (λ₁ₛₑ = 0.01528), the elastic-net model achieved a fivefold cross-validated area under the receiver operating characteristic (ROC) curve (AUC) of 0.989, representing an increase of 0.039 over the baseline model with RCS (TyG, UHR) alone (0.950). The coefficient paths (Fig. 2B) showed that, as regularization relaxed (i.e., decreasing log(λ)), SBP, DBP, and age were the first to deviate from zero, while the ALT/AST ratio and Scr stayed close to zero with stable signs. Figure 2C lists the selected features and their coefficients at λ₁ₛₑ, the nonzero predictors included SBP, DBP, age, ALT/AST ratio, and Scr, with standardized coefficients of 0.818, 0.387, 0.296, 0.026, and − 0.028, respectively. Despite small coefficients, the directions for ALT/AST ratio and Scr were clinically consistent. We used all five nonzero variables in subsequent models. Overall, the elastic-net analysis indicates that, besides the strong signals from TyG and UHR, simple clinical measures—especially blood pressure and age—offer additional discrimination, while maintaining a parsimonious and practical model.

### Comparison of machine learning algorithms and selection of the optimal model

**Fig. 3:**
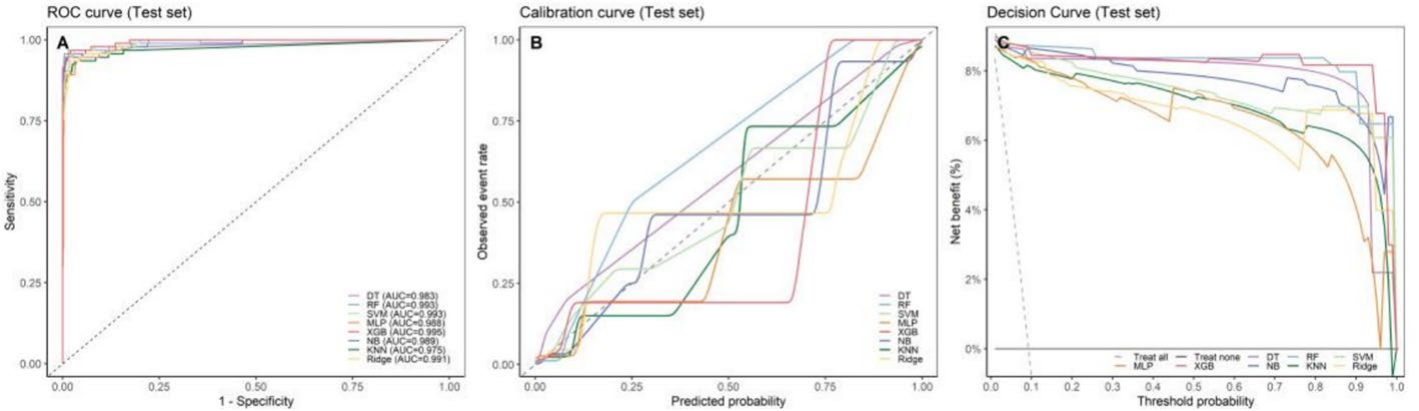
ROC, calibration curves, and DCA on the test set across candidate models. **A** ROC curves across candidate models on the test set. **B** Calibration curves on the test set after post hoc recalibration using the OOF calibrator fixed during development. The calibration curves were estimated using isotonic regression with Gaussian kernel smoothing. **C** DCA on the test set

**Fig. 4:**
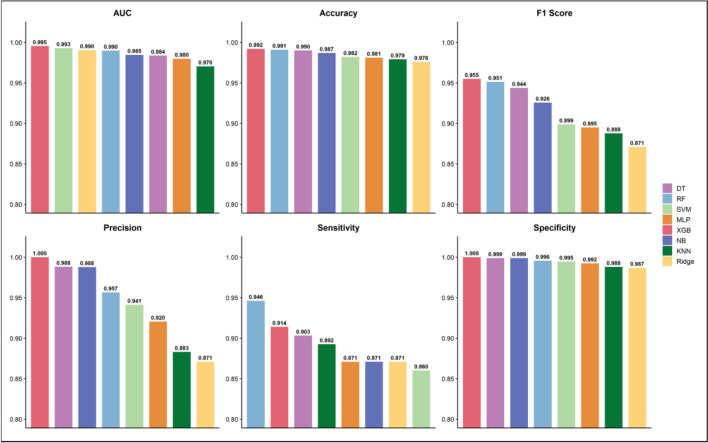
Comparison of key metrics on the test set at the F1-max operating point. This multi-panel bar chart summarizes model performance for AUC, Accuracy, F1 Score, Precision, Sensitivity, and Specificity. For each model, the decision threshold was selected on the test set to maximize F1 using post hoc calibrated probabilities; AUC is threshold independent and is shown for reference. Bar heights represent the metric values for each model

Using the prespecified predictors along with the clinical variables selected by elastic-net, we trained eight algorithms—decision tree (DT), random forest (RF), support-vector machine with radial-basis-function kernel (SVM-RBF), multilayer perceptron (MLP), extreme gradient boosting (XGBoost), naïve Bayes (NB), k-nearest neighbors (KNN), and ridge logistic regression—on the training set and assessed performance on the test set with the out-of-fold (OOF) calibrator fixed during model development. XGBoost demonstrated the best discrimination (AUC 0.995) and, at the F1-max threshold, the strongest overall classification performance with an F1 score (F1) of 0.955, accuracy (ACC) of 0.992, precision (PRE) of 1.000, specificity (SPE) of 1.000, and sensitivity (SEN) of 0.914. RF ranked second (AUC 0.990; SEN 0.946; ACC 0.991; F1 0.951) (Figs. 3A; 4). After post hoc calibration, Brier score and log loss decreased for all models; XGBoost achieved the lowest Brier score of 0.008 and log loss of 0.035, while maintaining an AUC of 0.995 (Supplementary Table S1). To further rigorously assess the alignment between predicted probabilities and observed outcomes, we calculated the expected calibration error (ECE). XGBoost exhibited an extremely low ECE of approximately 0.007 (Supplementary Table S1). In terms of calibration intercepts and slopes, results indicated that SVM-RBF was closest to ideal (intercept 0.021; slope 0.963), whereas XGBoost (intercept 0.667; slope 1.075) showed systematic underestimation, which was properly corrected through calibration (Fig. 3B; Supplementary Table S1). The observed deviations in the calibration curves within the intermediate probability ranges visually reflect the inherent data sparsity caused by the model's strong discriminative ability, a characteristic transparently captured by the isotonic regression with kernel smoothing. In DCA, across threshold probabilities of approximately 0.05–0.60, XGBoost and RF provided the highest or near-highest net benefit, outperforming the other models (Fig. 3C). Considering discrimination, probability calibration, and clinical net benefit collectively, we chose XGBoost as the primary model for subsequent analyses and external validation.

### External validation and post hoc recalibration on NHANES

**Fig. 5:**
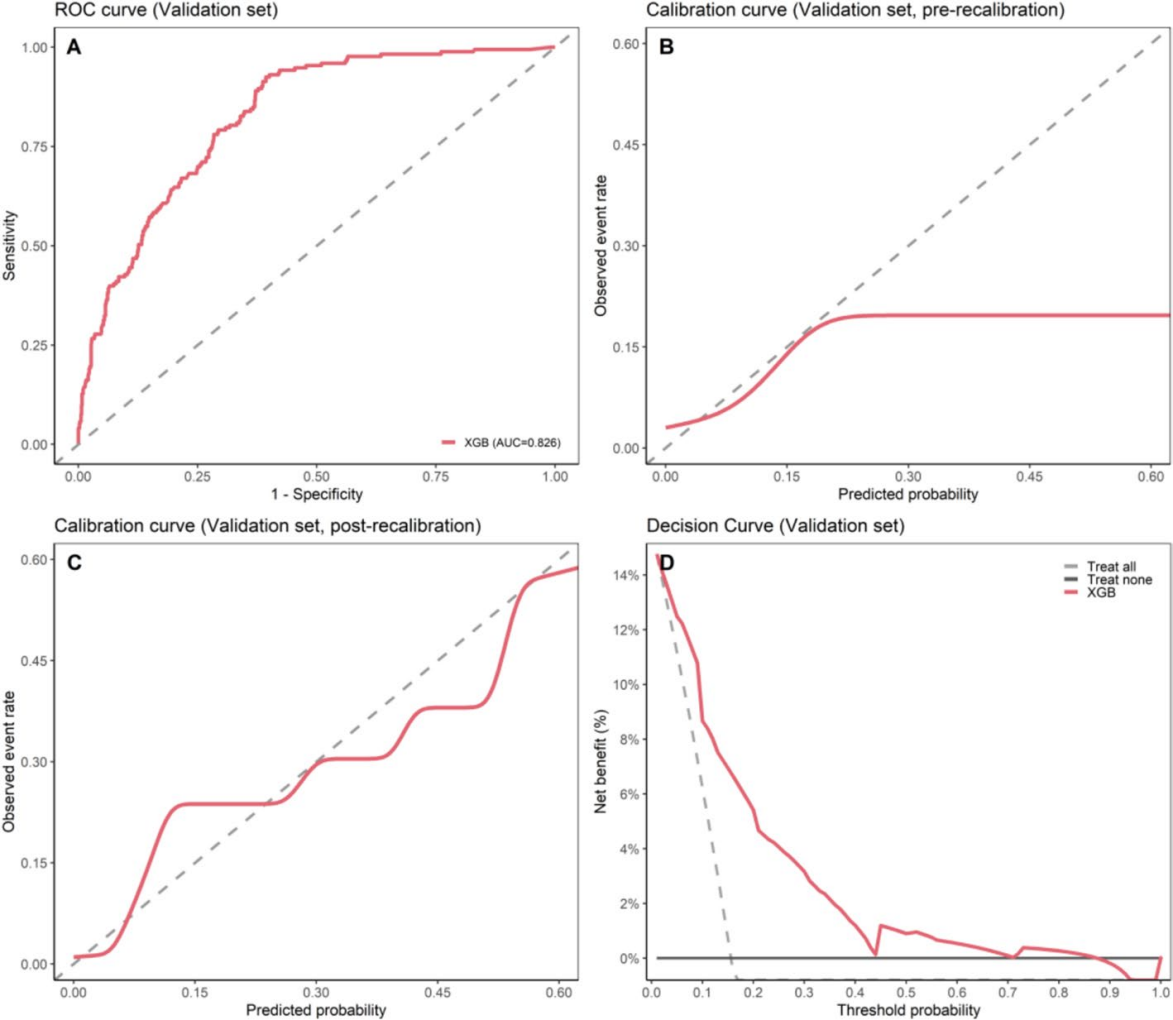
External validation of the XGBoost model on NHANES: ROC, calibration (pre/post), and DCA. **A** ROC curve on the validation set. **B** Calibration curve on the validation set before recalibration. **C** Calibration curve on the validation set after post hoc recalibration using isotonic regression. **D** DCA on the validation set

Using the locked XGBoost model, we selected the recalibration method on the NHANES calibration subset; isotonic regression was chosen because it achieved a lower Brier score (0.094). On the hold-out validation subset, discrimination was good before recalibration (AUC = 0.826) (Fig. 5A) and remained similar after applying the fixed calibrator (AUC = 0.810). Probability accuracy improved significantly: the Brier score decreased from 0.286 to 0.109, and the log loss from 1.253 to 0.388. Calibration of the recalibrated probabilities was acceptable, with a calibration intercept of 0.103 (95% CI =  − 0.085–0.290) and a calibration slope of 0.684 (95% CI = 0.540–0.829). Visually, the calibration curve moved from systematic miscalibration to close alignment with the 45° reference line after recalibration (Fig. 5B vs. C). In DCA, the model provided positive net benefit over treast-all and treat-none across threshold probabilities of approximately 0.02–0.30, peaking at lower thresholds (Fig. 5D). Overall, the externally recalibrated XGBoost model maintained useful discrimination and showed markedly improved calibration on NHANES.

### SHAP-based model explanation and biomarker-stratified feature profiles

**Fig. 6:**
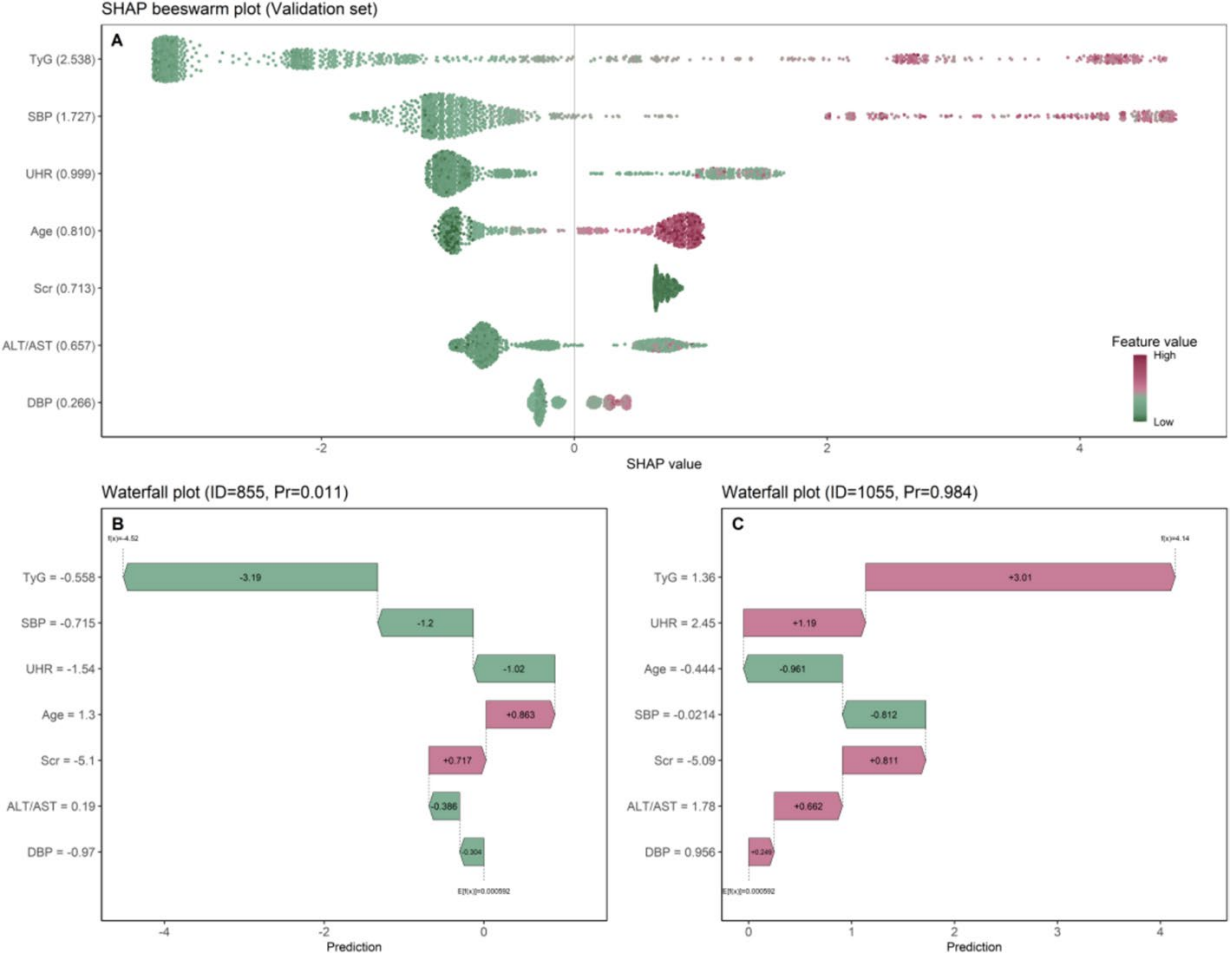
Global and individual-level SHAP explanations in the NHANES external validation set. **A** SHAP beeswarm plot ranking features by mean absolute SHAP value (global importance); numeric values in parentheses on the y-axis labels represent the mean absolute SHAP value for each feature; point color encodes the raw feature value (higher to lower). Positive SHAP values increase predicted risk; negative values decrease it. **B**–**C** Representative waterfall plots for one high-risk and one low-risk participant showing how each feature cumulatively shifts the prediction from the model baseline (expected value) to the individual probability

On the hold-out validation subset, SHAP analysis showed a clear and biologically plausible pattern of feature importance. In the global beeswarm plot (Fig. 6A), TyG had the highest mean |SHAP| (2.538), followed by SBP (1.727) and UHR (0.999), with age (0.810), Scr (0.713), and the ALT/AST ratio (0.657) contributing modestly, while DBP (0.266) had the smallest effect. Higher TyG and UHR values increased the prediction toward higher risk, while lower values shifted predictions toward lower risk. Waterfall plots exemplify these effects at the individual level: in a high-risk case (Fig. 6C), distinctively large positive contributions from TyG and UHR dominated the prediction, whereas in a low-risk case (Fig. 6B), lower values of TyG, UHR, and SBP cumulatively decreased the predicted risk.

**Fig. 7:**
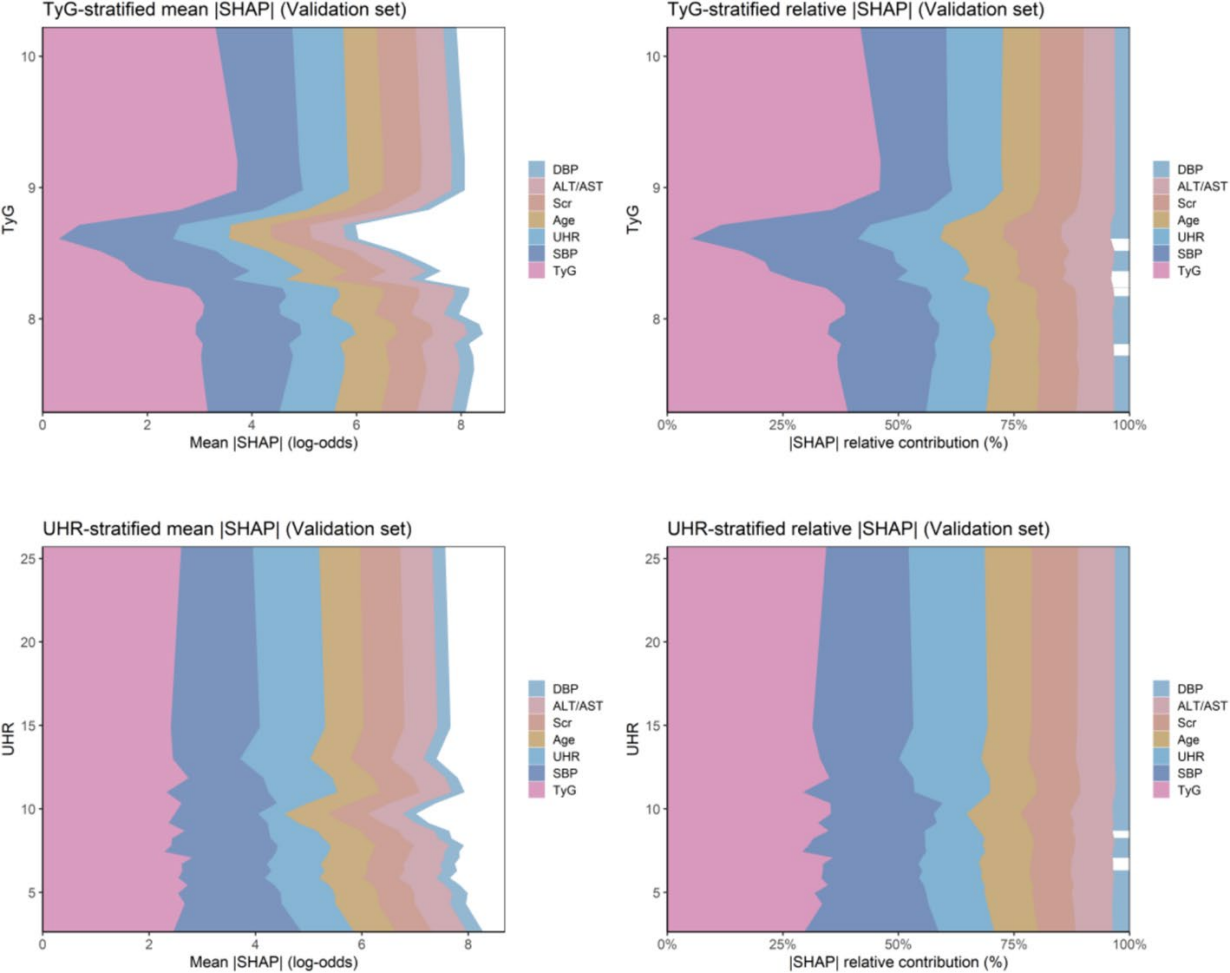
TyG- and UHR-stratified SHAP contribution profiles in the NHANES external validation set. Top row: TyG-stratified profiles; bottom row: UHR-stratified profiles. Left column: absolute contributions (stacked mean |SHAP| in log-odds) within each biomarker quantile bin. Right column: relative contributions (per-feature shares normalized to 100% within each bin)

To examine how feature influence varies across biomarker levels, we generated stratified |SHAP| profiles for TyG and UHR (Fig. 7). In terms of prediction intensity (absolute contributions), TyG exhibited a V-shaped pattern: it was highest in the lower and higher ranges of its spectrum but showed a distinct dip in the intermediate range. In contrast, UHR displayed a continuous profile where contribution increased linearly with rising values. In terms of feature composition (relative contributions), at lower TyG levels, the model’s predictive signal was predominantly shared among TyG, SBP, and UHR. However, as TyG levels exceeded the physiological threshold, its influence became more prominent. The UHR-stratified panels showed a similar but less pronounced trend: rising UHR was generally associated with increased relative contributions from TyG and UHR, accompanied by a relative decline in the SBP share, although collectively these three factors remained the dominant drivers regardless of UHR levels.

Together, these SHAP results confirm that the model’s decisions are driven primarily by the prespecified indices (TyG and UHR) with coherent, supporting contributions from routine clinical variables—especially SBP—and that the dominance of the biomarker signals becomes more pronounced at higher TyG/UHR levels.

## Discussion

In this cross-sectional study, we developed a screening model for lean MAFLD in a hospital health checkup group using simple, accessible indicators—TyG, UHR, and a small set of routine clinical variables. In the NHANES external validation set, the model demonstrated good discrimination, with an AUC of 0.826 before recalibration and 0.810 afterward. DCA indicated consistent positive net benefits across thresholds of about 0.02–0.30, peaking toward the lower end, which supports primary care triage needs. As a distinct phenotype, lean MAFLD is not uncommon: in a Taiwanese health checkup database of over 20,000 adults, Cheng et al. reported an overall MAFLD prevalence of 39.1%, with 12.3% being lean, emphasizing that this subtype is prevalent [18]. Cheah et al. also estimated a 1.94% prevalence of lean MAFLD in the general population; despite having a metabolic load comparable to overweight/obese MAFLD, lean MAFLD carries a higher risk of liver-related death [8]. However, during routine health exams and primary care, lean or low-BMI individuals are seldom considered high-risk, and screening awareness remains limited. Coupled with practical issues of imaging—cost, radiation exposure from CT, and limited capacity—risks are often overlooked. To address this gap, we propose a low-cost, automated, scalable, and explainable screening method based on TyG and UHR, supported by a minimal set of clinical variables. The model was carefully designed to prevent data leakage, recalibrated before external application, and accompanied by SHAP-based explanations to assist threshold-based triage and referral for confirmatory imaging.

MAFLD indicates systemic metabolic dysfunction centered in the liver and is part of the broader spectrum of metabolic-related cardiovascular and kidney syndromes. As the primary organ for managing glucose and lipids, the liver tends to deposit TG when mitochondrial β-oxidation is impaired due to an excess of fatty acids. Prior research shows a strong connection between lean MAFLD and IR [19, 20], with several studies reporting higher HOMA-IR levels in lean individuals compared to overweight or obese ones with MAFLD [20, 21]. Consistent with this, TyG is a useful surrogate marker for IR [22]. UHR, which indicates signals related to purine metabolism, inflammation, and lipid regulation, has shown independent links with MAFLD in a large Chinese health check cohort and NHANES [23, 24]. Our RCS analysis confirmed these findings: TyG has a notable nonlinear relationship with an inflection point around 8.5, while UHR shows a roughly linear association. In the validation set, SHAP beeswarm plots ranked TyG as the most influential factor, followed by SBP and UHR. Age, Scr, and the ALT/AST ratio had a moderate impact, with smaller but consistent signals from DBP. The stratified SHAP profiles revealed distinct patterns: TyG exhibited a V-shaped intensity with a dip in the intermediate physiological range, where the risk signal was weakest, corroborating the nonlinear threshold effect observed in our RCS analysis, whereas UHR showed a continuous linear contribution. This multi-dimensional pattern—highlighting IR, hemodynamic stress, purine/inflammation, and lipid balance—aligns with the pathophysiology of the lean phenotype and supports personalized risk communication and lifestyle advice after screening.

Regarding predictor selection, beyond the prespecified TyG and UHR indices, we used elastic-net regression to screen routine clinical variables, thereby enhancing both model reliability and clinical interpretability. This approach yielded clear performance gains: at the optimal regularization parameter (λ₁ₛₑ = 0.01528), the fivefold cross-validated AUC increased from 0.950 with TyG and UHR alone to 0.989 with the expanded set. This improvement demonstrates that the model achieved superior discrimination while maintaining simplicity. Furthermore, the inclusion of SBP, DBP, age, the ALT/AST ratio, and Scr is biologically plausible and aligns well with the elevated cardiometabolic risk profile characteristic of the lean MAFLD phenotype. Building on this optimized predictor set and the comparative performance of candidate models in the development cohort, our final selection of XGBoost was driven by a strategic trade-off between strict calibration metrics and overall clinical utility. Although the RF achieved a slightly better Brier score, XGBoost offered the optimal balance for a screening tool, combining high discrimination (AUC 0.995) with minimized prediction error (log loss 0.0349). Most importantly, DCA favored XGBoost, which provided the highest net benefit across a broad range of clinically plausible thresholds (0.05–0.60), thereby maximizing its value for primary care triage.

For external validation, NHANES data were divided equally into calibration and hold-out subsets. Recalibration significantly lowered the Brier score and log loss with minimal impact on AUC, showing that cross-domain transfer mainly affects probability scaling rather than ranking. Due to differences in steatosis detection methods (ultrasound vs. CAP), lean thresholds (< 23 vs. < 25 kg/m^2^), and population structure, a predict-then-recalibrate approach is necessary. In practice, deploying the model at new sites can rely on lightweight recalibration using a small labeled sample to restore calibration and stabilize threshold interpretation without re-creating the model. Based on DCA, thresholds around 0.02–0.30 are appropriate for triage under typical resource constraints: at the time of lab reporting, the laboratory information systems (LIS) can automatically calculate TyG/UHR and the risk score with a brief SHAP explanation; individuals above a set threshold can be referred for liver ultrasound or CAP, while those in a watch zone can undergo lifestyle management followed by re-evaluation.

Compared to prior studies, our approach differs in several ways. Zhu et al. defined lean MAFLD using BMI < 25 kg/m^2^ and developed a nomogram with multivariable logistic regression [25]; we used region-specific BMI thresholds and a machine learning framework, selecting the best algorithm through systematic comparison. Cui et al. proposed a nomogram for lean inpatients with type 2 diabetes but did not test its generalizability on external data [26]; by training in a Chinese hospital health checkup cohort and validating in NHANES, our model shows stronger transportability and scalability. This study still has limitations. The cross-sectional design limits inference on incidence and progression. Differences between health checkup populations and clinical or inpatient cohorts may introduce selection and spectrum bias. Outcomes were defined by ultrasound in development and by CAP in external validation, so threshold and sensitivity differences can cause domain shift; recalibration reduced but did not eliminate this issue, and confirmation with additional imaging standards is needed. Region-specific lean thresholds (< 23 vs. < 25 kg/m^2^) require local adaptation and sensitivity analyses. Finally, some key anthropometrics, such as waist circumference, were not systematically recorded in the development cohort and may underestimate metabolic burden. Future work will focus on multi-center dynamic recalibration, subgroup fairness evaluation, and prospective impact studies to establish real-world utility.

## Conclusions

In this study, we developed a simple yet interpretable screening model for lean MAFLD using TyG, UHR, and some routine clinical variables. Systematic comparison led us to select XGBoost, which showed excellent discrimination and calibration internally and maintained good performance on NHANES data. Post hoc recalibration significantly improved probability accuracy while preserving the rank order. DCA identified a practical threshold range of approximately 0.02–0.30 with consistent net benefits, supporting its use as an automated triage tool in health checkups and primary care. RCS and SHAP analyses aligned with biological understanding, revealing a nonlinear effect of TyG and an approximately linear effect of UHR, with SBP, age, and other variables adding additional risk factors. Differences in steatosis detection methods, region-specific lean cutoff points, and cohort diversity highlight the need for a predict-then-recalibrate approach for local implementation.

## Methods

### Data sources

This cross-sectional study used routinely collected health checkup data from the Health Management Center of Huai’an First People’s Hospital, affiliated with Nanjing Medical University in Huai’an, Jiangsu, China. Records for model development and internal testing were retrospectively obtained from hospital information systems, LIS, and picture archiving and communication systems from January 2023 to January 2025. For individuals with multiple visits during the study period, only the first eligible visit was included. To ensure reproducibility, a de-identified analysis dataset was finalized on March 19, 2025, before modeling.

For external recalibration and validation, we used publicly available data from NHANES 2017–2020, managed by the U.S. Centers for Disease Control and Prevention. One-to-one variable harmonization—covering feature names, coding, and unit conversions—between the hospital cohort and NHANES is explained in Supplementary Table S2.

All data were de-identified before analysis. The hospital cohort protocol received approval from the Medical Ethics Committee of Huai’an First People’s Hospital, affiliated with Nanjing Medical University (approval number KY-2025-123-01). Written informed consent was waived due to the retrospective nature, minimal risk, and use of de-identified data. NHANES participants gave written informed consent under protocols approved by the National Center for Health Statistics Institutional Review Board, and this secondary analysis of de-identified, publicly available data did not require additional institutional review.

### Participants

#### Hospital cohort

Inclusion criteria were as follows: (1) age ≥ 18 years; (2) any sex; (3) hepatic ultrasound performed; and (4) BMI available without missing values. Exclusion criteria included the following: (1) known viral, drug-induced, or autoimmune hepatitis; (2) active consumptive conditions such as tuberculosis or malignancy; (3) previous liver surgery; and (4) complete absence of all metabolic syndrome (MetS) component variables, which prevented adjudication of metabolic dysfunction. After applying these criteria, *N* = 3346 individuals were eligible.

#### External dataset: NHANES 2017–2020

Inclusion criteria were as follows: (1) age ≥ 18 years; (2) any sex; (3) availability of VCTE with CAP; and (4) BMI data available. The exclusion criterion was a complete absence of all MetS component variables. A total of *N* = 2216 participants qualified for external analysis. Criteria for the external dataset differed slightly from the hospital cohort to accommodate differences in data availability (specifically, the use of VCTE instead of ultrasound and the lack of comprehensive medical records for strict exclusions).

#### Lean phenotype and MAFLD case definition

To reflect region-specific standards, we defined the lean phenotype a priori as BMI < 23 kg/m^2^ in the Chinese hospital cohort and < 25 kg/m^2^ in the U.S. NHANES cohort [11, 18]. MAFLD was defined as hepatic steatosis plus metabolic dysfunction following current consensus criteria [3]. Steatosis ascertainment: ultrasound-detected fatty liver in the hospital cohort or CAP ≥ 238 dB/m measured by VCTE in NHANES [27, 28]. Metabolic dysfunction included any of the following—(1) overweight or obesity, defined as BMI ≥ 23 kg/m^2^ in the hospital cohort and ≥ 25 kg/m^2^ in NHANES; (2) type 2 diabetes; or (3) at least two abnormal MetS components from SBP ≥ 130 mmHg or DBP ≥ 85 mmHg; HDL-C < 1.0 mmol/L in men or < 1.3 mmol/L in women; TG ≥ 1.7 mmol/L; FBG ≥ 5.6 mmol/L; or HbA1c ≥ 5.7% [11]. Waist circumference was not used to determine metabolic dysfunction, as the study population was mostly lean, and for workflow and feasibility reasons, waist measurements were not routinely collected in the health screening setting. In analyses limited to the lean phenotype, the overweight/obesity criterion was not applied; thus, lean MAFLD required steatosis, BMI below the region-specific lean threshold, and either type 2 diabetes or at least two abnormal MetS components from the list above. In NHANES, additional biomarkers—including the HOMA-IR and CRP—were available; however, these measures were not routinely collected in the hospital cohort and were not used for MAFLD classification or as primary predictors in the screening model.

#### Imaging acquisition and quality control

All ultrasound examinations in the hospital cohort followed a standardized workflow. Participants were scanned in either the supine or left lateral decubitus position using a Samsung HS60 color Doppler ultrasound system with a convex array transducer (CA1–7AD). For each participant, three standardized hepatic images were obtained: (1) right intercostal oblique view at the first porta hepatis; (2) right subcostal oblique view at the first porta hepatis; and (3) right subcostal oblique view at the second porta hepatis. Images were captured with the screen frozen and saved in Digital Imaging and Communications in Medicine format. All ultrasound images underwent secondary review by two senior sonographers, with discrepancies resolved by consensus. Ultrasound steatosis was defined by increased hepatic echogenicity relative to the renal cortex, along with vascular blurring and posterior attenuation [29].

#### NHANES transient elastography quality criteria

Standard reliability thresholds were required: at least 10 valid measurements and an interquartile range-to-median ratio for liver stiffness ≤ 0.30; CAP measurements failing manufacturer quality flags were excluded.

#### Participant flow

**Fig. 8:**
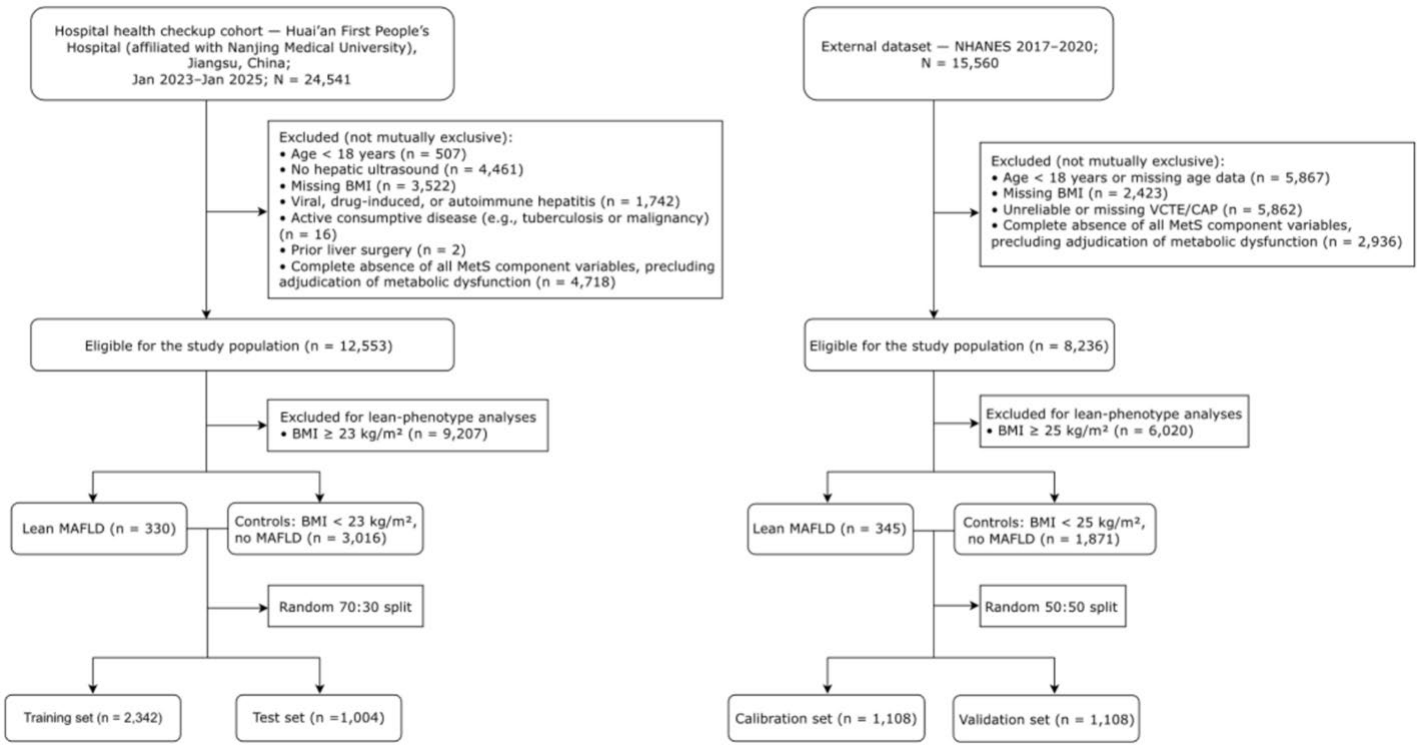
STROBE flow diagram of participant selection

A STROBE-style flow diagram (Fig. 8) summarizes screening and exclusions (with counts for each reason), the final numbers included (3346 in the hospital cohort and 2216 in NHANES), and the subsequent 70:30 internal split of the hospital cohort and 50:50 split of NHANES.

### Predictors

We prespecified two index predictors: TyG and UHR. TyG was calculated as ln [TG (mg/dL) × FBG (mg/dL)/2] [30]. When TG or FBG were reported in mmol/L, values were converted using TG (mg/dL) = 88.57 × TG (mmol/L) and FBG (mg/dL) = 18 × FBG (mmol/L). UHR was calculated as serum uric acid (UA, mg/dL)/HDL-C (mg/dL) × 100 [16, 31]. Where required, units were harmonized using the following conversion factors: UA, 1 mg/dL = 59.48 µmol/L; HDL-C, 1 mmol/L = 38.67 mg/dL. All calculations were performed after unit harmonization.

To assess their independent diagnostic value and potential threshold effects, we fitted multivariable logistic regression models to the entire hospital cohort: TyG only; UHR only; both TyG and UHR; and an extended model that included clinical covariates selected via data-driven elastic-net regularization. In the final model, TyG and UHR were modeled using RCS with four knots placed at data-driven quantiles (specifically, standard sample percentiles), implemented in the rms package (v6.8.2). We report likelihood-ratio test *P* values for the overall association and for nonlinearity.

Beyond TyG and UHR, routinely collected variables from health checkups and primary care were considered as candidate supplementary predictors. These were screened and selected using an elastic-net logistic regression model (see “Elastic-net–based augmentation of clinical predictors” below).

### Model development

#### Data split and leakage control

For internal evaluation, the hospital health checkup cohort was randomly divided into training and test sets with outcome stratification (using random seed 20250812) at a 70:30 ratio. All preprocessing, candidate filtering, hyperparameter tuning, resampling, and OOF calibration were performed exclusively within the training set and its resampling folds. The test set was kept for a final evaluation to prevent information leakage.

#### Preprocessing and class imbalance

A standardized preprocessing pipeline was applied to all candidate models: (1) median imputation (as shown in Supplementary Figure S1, the final selected predictors exhibited negligible missing rates; thus, the median method was deemed robust and unlikely to bias variance estimates); and (2) z-score normalization. Class imbalance was managed carefully within resampling to prevent information leakage: when the number of minority-class samples per fold was ≥ 2, we used the synthetic minority over-sampling technique; otherwise, we employed random upsampling [32].

#### Masking and correlation filtering of candidates

To prevent proxy or duplicate information with TyG or UHR, variables explicitly representing or directly derived from TG, FBG, HbA1c, UA, HDL-C, and their ratios or indices were masked beforehand using name-based rules. Remaining numeric candidates were screened by Pearson correlation against TyG and UHR separately; any variable with |*r*|≥ 0.70 with either was excluded.

#### Elastic-net-based augmentation of clinical predictors

Within the training set, we fitted a binomial elastic-net logistic regression (glmnet, v4.1.8) to identify additional clinical predictors. TyG and UHR were modeled with four-knot RCS and left unpenalized, whereas the remaining candidates were standardized and penalized with a combined L1/L2 penalty (α = 0.5). The baseline model contained only RCS(TyG) and RCS(UHR). We selected λ₁ₛₑ using stratified fivefold cross-validation, with the AUC as the selection metric. All predictors with nonzero coefficients at λ₁ₛₑ were carried forward to subsequent modeling.

#### Candidate learners and engines

Eight algorithms were implemented via the tidymodels framework: DT (rpart, v4.1.23), RF (ranger, v0.17.0), SVM-RBF (kernlab, v0.9.33), MLP (nnet, v7.3.19), XGBoost (xgboost, v1.7.8.1), NB (klaR, v1.7.3), KNN (kknn, v1.4.1), and ridge logistic regression as a baseline comparator (glmnet, v4.1.8). Hyperparameter tuning and resampling were confined to the training set using fivefold stratified cross-validation to maximize average precision (AP) and prevent information leakage. All analyses were performed in R 4.4.1 on Windows 10 × 64; the complete software environment and package versions are summarized in Supplementary Table S3.

#### Optimization target and OOF calibration

The primary optimization metric was AP [33]. We tracked AUC, log loss, and the Brier score as secondary metrics. Metrics were calculated using yardstick (v1.3.2) and pROC (v1.18.5). For each tuned workflow, we aggregated the OOF predicted probabilities and compared Platt scaling with isotonic regression [34]; the calibrator with the lower OOF Brier score was chosen for all subsequent evaluations.

#### Test set evaluation and decision analysis

On the test set, using calibrated probabilities, we reported the AUC, Brier score, and log loss, and assessed calibration via the intercept, slope, and ECE. The results were summarized in Supplementary Table S1. The optimal threshold was identified by maximizing the F1 score via a grid search (0.01–0.99). At this threshold, we calculated the ACC, PRE, SEN, SPE, and F1, and visualized them in a multi-panel bar chart. Clinical utility was evaluated with DCA against treat-all and treat-none strategies.

### External validation and SHAP-based explainability

#### Data split and preprocessing

The NHANES sample was randomly divided 50:50 into a calibration subset and a hold-out validation subset, stratified by the MAFLD outcome (random seed 20250818). Predictors mirrored those in the training set and were derived from harmonized biochemical inputs according to predefined rules. To prevent information leakage, we reused only the training-derived preprocessing—median imputation and z-score normalization—by applying parameters learned from the training data to NHANES; no statistics were re-estimated, and no model hyperparameters were tuned.

#### Prediction and post hoc recalibration

Using the locked final model, we first generated raw predicted probabilities on the calibration subset. We then conducted post hoc recalibration by comparing Platt scaling and isotonic regression, choosing the method with the lower Brier score on the calibration subset. Probabilities were clipped only to prevent numerical extremes. The selected mapping was fixed and applied to the hold-out validation subset; the model structure and hyperparameters remained unchanged throughout NHANES.

#### External validation and visualization

Using the frozen calibrator together with the locked model, we obtained calibrated probabilities for the hold-out validation subset. External performance is summarized with four figures: (1) the ROC curve with AUC; (2) the calibration curve using raw probabilities versus observed event rates; (3) the calibration curve using recalibrated probabilities versus observed event rates; and (4) the DCA based on the calibrated probabilities.

#### SHAP-based explainability

We computed Shapley Additive Explanations (SHAP) values on the hold-out validation subset [35]. A beeswarm plot ranked features globally by mean absolute SHAP values and conveyed directionality through point color, while individual waterfall plots for one high-risk and one low-risk participant illustrated how features shifted predictions from the baseline to each estimate. To examine biomarker-dependent patterns, the cohort was divided into 20 quantiles by TyG or UHR; for each bin, we generated an absolute profile—stacked by-feature mean absolute SHAP values against the bin median of TyG or UHR—and a relative profile in which per-feature contributions were normalized to 100%, revealing shifts in dominant drivers across biomarker levels.

## Supplementary Information

Additional file 1: **Figure. S1** Distribution of missing values across study variables in the hospital checkup and NHANES cohorts. Top panel: Number of missing values for each variable in the hospital health checkup cohort. Bottom panel: Number of missing values for each variable in the NHANES 2017–2020 cohort. Variables are ranked by missing count.

Additional file 2: **Table. S1** Uncalibrated vs. post hoc calibrated metrics on the test set across candidate models.

Additional file 3: **Table. S2** Variable harmonization between the hospital checkup cohort and NHANES 2017–2020: field mapping and common analysis units.

Additional file 4: **Table. S3** Software and reproducibility.

## Data Availability

The external validation datasets analyzed during the current study are available in the NHANES database (https://wwwn.cdc.gov/nchs/nhanes/default.aspx). Hospital cohort data from the development stage can be obtained by contacting the corresponding author, and external validation data is also accessible.

## Abbreviations

ACC: Accuracy
ALB: Albumin
ALP: Alkaline phosphatase
ALT: Alanine aminotransferase
ALT/AST: Alanine/aspartate aminotransferase ratio
AP: Average precision
AST: Aspartate aminotransferase
AUC: Area under the ROC curve
BMI: Body mass index
BUN: Blood urea nitrogen
CAP: Controlled attenuation parameter
CK: Creatine kinase
CRP: C-reactive protein
CT: Computed tomography
Cys-C: Cystatin C
DBP: Diastolic blood pressure
DCA: Decision curve analysis
DT: Decision tree
ECE: Expected calibration error
F1: F1 score
FBG: Fasting blood glucose
FT3: Free triiodothyronine
FT4: Free thyroxine
GGT: γ-Glutamyltransferase
GLB: Globulin
HBDH: Hydroxybutyrate dehydrogenase
HDL-C: High-density lipoprotein cholesterol
HOMA-IR: Homeostatic model assessment of insulin resistance
HbA1c: Glycated hemoglobin A1c
IR: Insulin resistance
KNN: K-nearest neighbors
LDH: Lactate dehydrogenase
LDL-C: Low-density lipoprotein cholesterol
LIS: Laboratory information systems
Lp(a): Lipoprotein(a)
MAFLD: Metabolic dysfunction–associated fatty liver disease
MetS: Metabolic syndrome
MLP: Multilayer perceptron
MRI: Magnetic resonance imaging
NAFLD: Nonalcoholic fatty liver disease
NB: Naïve Bayes
NHANES: National Health and Nutrition Examination Survey
OOF: Out-of-fold
PAB: Prealbumin
PRE: Precision
RBC: Red blood cell count
RCS: Restricted cubic splines
RF: Random forest
ROC: Receiver operating characteristic
SBP: Systolic blood pressure
SEN: Sensitivity (recall)
SHAP: Shapley Additive Explanations
SPE: Specificity
SVM/SVM-RBF: Support-vector machine with radial-basis-function kernel
Scr: Serum creatinine
TC: Total cholesterol
TG: Triglycerides
TSH: Thyroid-stimulating hormone
TyG: Triglyceride–glucose index
UA: Uric acid
UHR: Uric acid–to–HDL cholesterol ratio
VCTE: Vibration-controlled transient elastography
WBC: White blood cell count
XGB/XGBoost: EXtreme Gradient Boosting
