# Supplementary material for: A TyG–UHR-based machine learning model for screening lean MAFLD: development and external validation
Source: Biomed Eng Online. 2026 May 11;25:86. doi: 10.1186/s12938-026-01585-8 (PMC13330354; doi:10.1186/s12938-026-01585-8)
Supplement: Supplementary file 1 — Additional file 1: Figure. S1 Distribution of missing values across study variables in the hospital checkup and NHANES cohorts. Top panel: Number of missing values for each variable in the hospital health checkup cohort. Bottom panel: Number of missing values for each variable in the NHANES 2017–2020 cohort. Variables are ranked by missing count. [file 12938_2026_1585_MOESM1_ESM.docx]

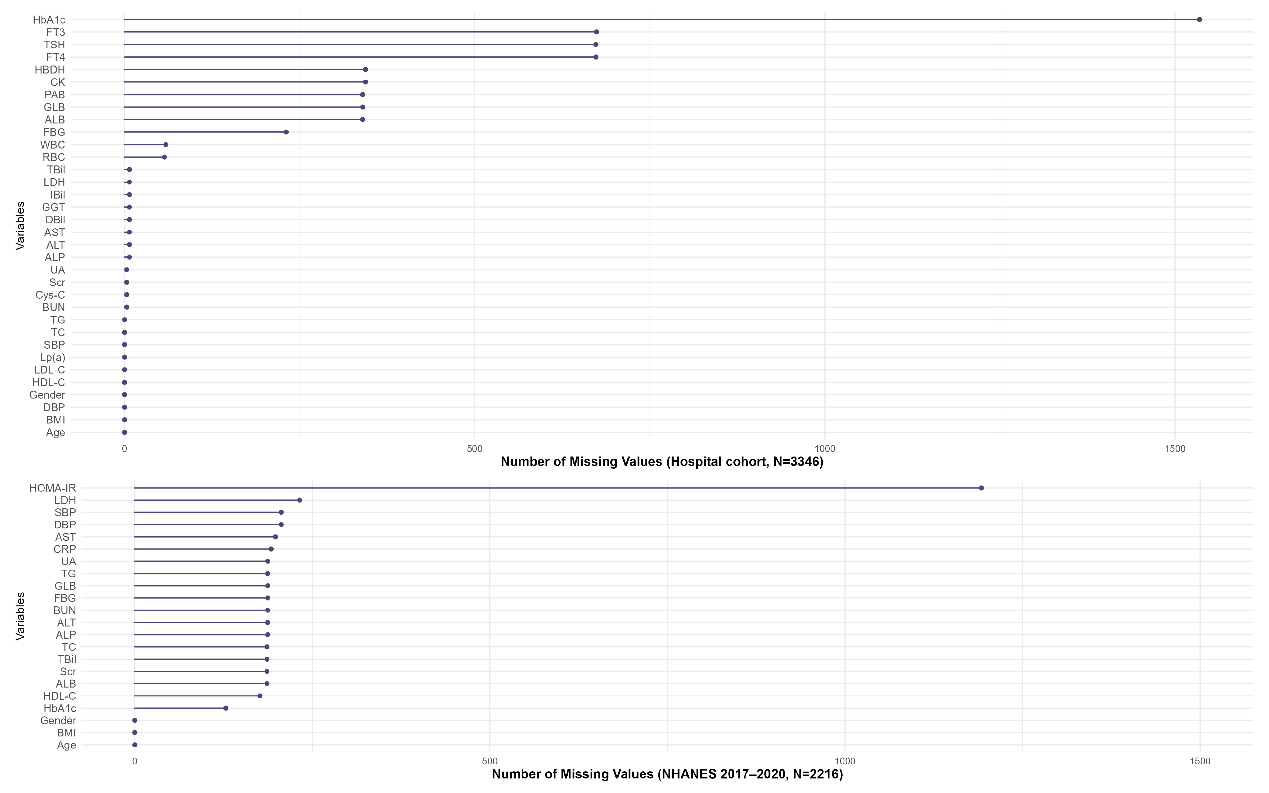


**Fig.S1** Distribution of missing values across study variables in the hospital checkup and NHANES cohorts. ***Top panel****: Number of missing values for each variable in the hospital health checkup cohort.* ***Bottom panel****: Number of missing values for each variable in the NHANES 2017–2020 cohort. Variables are ranked by missing count.*
