# Supplementary material for: A TyG–UHR-based machine learning model for screening lean MAFLD: development and external validation
Source: Biomed Eng Online. 2026 May 11;25:86. doi: 10.1186/s12938-026-01585-8 (PMC13330354; doi:10.1186/s12938-026-01585-8)
Supplement: Supplementary file 2 — Additional file 2: Table. S1 Uncalibrated vs. post hoc calibrated metrics on the test set across candidate models. [file 12938_2026_1585_MOESM2_ESM.docx]

| **Model** | **Brier**  **(raw)** | **Brier (calibrated)** | **Log Loss**  **(raw)** | **Log Loss (calibrated)** | **AUC**  **(raw)** | **AUC (calibrated)** | **Intercept** | **Slope** | **ECE (calibrated)** |
| --- | --- | --- | --- | --- | --- | --- | --- | --- | --- |
| XGBoost | 0.012007 | 0.008267 | 0.046963 | 0.034912 | 0.995302 | 0.995444 | 0.666917 | 1.075321 | 0.006583 |
| SVM-RBF | 0.021027 | 0.013591 | 0.073923 | 0.047522 | 0.992942 | 0.992924 | 0.020823 | 0.963368 | 0.004076 |
| Ridge | 0.019979 | 0.018608 | 0.079427 | 0.063595 | 0.990794 | 0.990481 | -0.042610 | 0.884218 | 0.011624 |
| Random Forest | 0.011081 | 0.007623 | 0.055138 | 0.057009 | 0.992611 | 0.989843 | 0.286980 | 0.835285 | 0.005050 |
| Naive Bayes | 0.033024 | 0.010765 | 0.120151 | 0.087715 | 0.989436 | 0.984650 | -0.635240 | 0.590613 | 0.004039 |
| Decision Tree | 0.014101 | 0.009498 | 0.056609 | 0.046776 | 0.983192 | 0.983682 | 1.279760 | 1.224837 | 0.005786 |
| Neural Net | 0.087051 | 0.018441 | 0.344890 | 0.117140 | 0.988209 | 0.979923 | -0.337280 | 0.576293 | 0.014182 |
| KNN | 0.026857 | 0.016715 | 0.160377 | 0.178979 | 0.974960 | 0.970362 | -0.967490 | 0.370830 | 0.004171 |

**Table S1** Uncalibrated vs. post hoc calibrated metrics on the test set across candidate models.
