# Supplementary material for: A TyG–UHR-based machine learning model for screening lean MAFLD: development and external validation
Source: Biomed Eng Online. 2026 May 11;25:86. doi: 10.1186/s12938-026-01585-8 (PMC13330354; doi:10.1186/s12938-026-01585-8)
Supplement: Supplementary file 4 — Additional file 4: Table. S3 Software and reproducibility. [file 12938_2026_1585_MOESM4_ESM.docx]

**Table S3 Software and reproducibility**

**A. System and R environment**

| **Item** | **Value** |
| --- | --- |
| sysname | Windows |
| release | 10 x64 |
| version | build 26100 |
| machine | x86-64 |
| platform | x86_64-w64-mingw32 |
| R.version | R version 4.4.1 (2024-06-14 ucrt) |

**B. Key R packages and versions**

| **Package** | **Version** |
| --- | --- |
| Hmisc | 5.2.0 |
| dials | 1.4.1 |
| discrim | 1.0.1 |
| doFuture | 1.1.2 |
| dplyr | 1.1.4 |
| finetune | 1.2.1 |
| future | 1.67.0 |
| glmnet | 4.1.8 |
| hardhat | 1.4.1 |
| kernlab | 0.9.33 |
| kknn | 1.4.1 |
| klaR | 1.7.3 |
| nnet | 7.3.19 |
| pROC | 1.18.5 |
| parsnip | 1.3.2 |
| ranger | 0.17.0 |
| readr | 2.1.5 |
| recipes | 1.3.1 |
| rms | 6.8.2 |
| rpart | 4.1.23 |
| rsample | 1.3.1 |
| themis | 1.0.3 |
| tibble | 3.2.1 |
| tidymodels | 1.3.0 |
| tune | 1.3.0 |
| workflows | 1.2.0 |
| xgboost | 1.7.8.1 |
| yardstick | 1.3.2 |
